# Trastuzumab Resistance: Role for Notch Signaling

**DOI:** 10.1100/tsw.2009.166

**Published:** 2009-12-16

**Authors:** Kinnari Mehta, Clodia Osipo

**Affiliations:** ^1^ Molecular Biology Program, Cardinal Bernardin Cancer Center, Loyola University Medical Center, Maywood, IL, USA; ^2^ Department of Pathology, Oncology Institute, Cardinal Bernardin Cancer Center, Maywood, IL, USA; ^3^ Molecular and Cellular Biochemistry Program, Cardinal Bernardin Cancer Center, Loyola University Medical Center, Maywood, IL, USA

**Keywords:** ErbB-2, trastuzumab, Notch-1, GSI, breast cancer

## Abstract

Epidermal growth factor receptor-2 (ErbB-2/HER2) is a potent breast oncogene that has been shown to be amplified in 20% of breast cancers. Overexpression of ErbB-2 predicts for aggressive tumor behavior, resistance to some cytotoxic and antihormonal therapies, and poor overall survival. Trastuzumab, the humanized, monoclonal antibody directed against ErbB-2 has shown tremendous efficacy and improved overall survival for women when combined with a taxane-based chemotherapy. However, resistance to trastuzumab remains a major concern, most notably in women with metastatic breast cancer. Numerous mechanisms that include overexpression of alternate receptor tyrosine kinases and/or loss of critical tumor suppressors have been proposed in the last several years to elucidate trastuzumab resistance. Here we review the many possible mechanisms of action that could contribute to resistance, and novel therapies to prevent or reverse the resistant phenotype. Moreover, we provide a critical role for Notch signaling cross-talk with overlapping or new signaling networks in trastuzumab-resistant breast.

Human epidermal growth factor receptor-2 (ErbB-2) is a type I transmembrane receptor tyrosine kinase. ErbB-2 and other family members (EGFR, ErbB-3, and ErbB-4) contain an extracellular ligand-binding domain, a transmembrane domain, and an intracellular tyrosine kinase domain. Receptor dimerization is required for activation and subsequent downstream signaling, mediated primarily by the phosphoinositide 3-kinase (PI3K) and mitogen-activated protein kinase (MAPK) pathways. Homodimerization or heterodimerization activate the intracellular tyrosine kinase domain initiating autophosphorylation of tyrosine residues within the cytoplasmic tail, which then triggers PI3K and MAPK signaling pathways, resulting in cell survival and proliferation[1,2,3]. The crystal structure of the extracellular domain of ErbB receptors identified four subdomains that are critical for ligand binding in the case of EGFR, ErbB-3, and ErbB-4 (subdomains I: L1 and subdomain III: L2), and receptor dimerization (cysteine-rich subdomains II: S1 and IV: S2)[1]. The crystal structure of ErbB-2 revealed that its ligand-binding domain might already be in a "ligand bound" conformation. Thus, this might explain why no ligand has yet been identified for ErbB-2[1].

ErbB-2–positive breast cancers, which account for 20–30% of all breast cancers, exhibit gene amplification and protein overexpression of ErbB-2. Women diagnosed with this subtype are characterized by an aggressive phenotype and decreased overall survival. The ErbB-2 protein is expressed in all ErbB-2–amplified tumor cells, both at the primary and metastatic sites[4]. Currently, trastuzumab, a recombinant, humanized, monoclonal antibody directed against the extracellular juxtadomain of ErbB-2[5], is a ErbB-2–targeted therapy approved by the FDA for the treatment of ErbB-2–positive breast cancer. The exact mechanism by which trastuzumab inhibits ErbB-2 signaling is not yet fully understood. However, some of the possible mechanisms include inhibition of receptor-receptor interaction, endocytic-mediated receptor down-regulation, blocking cleavage of the extracellular domain of the receptor, and activation of antibody-dependent cell-mediated cytotoxicity[6]. In the treatment of ErbB-2–positive breast cancer, trastuzumab showed significant efficacy in the adjuvant settings with an overall response rate (ORR) of 26%, which increased to about 90% when combined with chemotherapeutic agents[4,7,8,9]. However, despite its proven efficacy and dramatic effects on survival, 20–50% of women with ErbB-2–positive, metastatic breast cancer exhibit intrinsic resistance, which means that they do not respond to trastuzumab[10,11]. Furthermore, 10–15% of the women treated with trastuzumab plus chemotherapy in the adjuvant setting developed acquired resistance within the first year, which means that they initially responded to trastuzumab, but became resistant during treatment[7,12]. Lapatinib, a dual EGFR/ErbB-2 tyrosine kinase inhibitor, is approved for the treatment of ErbB-2–positive breast cancer that has advanced during or after trastuzumab treatment[13,14,15]. Recently, in a phase III clinical trial, lapatinib plus paclitaxel showed benefit as a first-line treatment of metastatic breast cancer[16]. Future combination studies including trastuzumab and lapatinib are now being evaluated in the metastatic setting to assess clinical benefit and progression-free survival vs. using trastuzumab as a single agent. However, resistance to lapatinib occurs in cell culture models *in vitro*[17] and within the first year of treatment. Thus, understanding the mechanisms responsible for resistance to ErbB-2–targeted therapies is critical to identify novel targets to prevent and/or reverse the resistant phenotype that is responsible for disease progression and the majority of deaths.

## POTENTIAL MECHANISMS RESPONSIBLE FOR RESISTANCE TO TRASTUZUMAB

Extensive efforts have been made to understand the exact mechanism of acquired resistance to trastuzumab using cell culture models. In these models, cells are treated continuously with trastuzumab until the growth is no longer inhibited and the cells continue to proliferate even in the presence of trastuzumab. Preclinical studies propose numerous possible molecular mechanisms by which breast cancer cells evade trastuzumab therapy. Identification of signaling pathways that are responsible for resistance (summarized in Table 1) has allowed and will allow for the development of novel therapeutic agents. Each of the novel agents, either alone or when combined with trastuzumab, could potentially open new horizons that may help to replace trastuzumab or increase the responsiveness to trastuzumab to prevent and/or reverse resistance.

### Role for Receptor Tyrosine Kinases

ErbB family members are functionally redundant. Dimerization, transphosphorylation, and activation of some redundant downstream signaling molecules are important functions of ErbB signaling pathways. Despite the proven ability of trastuzumab to inhibit ErbB-2 phosphorylation, it rarely blocks the dimerization of ErbB-2 with other ErbB family members. Recently, elevated EGFR and HER3 expression have been reported when ErbB-2–overexpressing breast cancer cells (T47D, UACC812, UACC893, MDA-MB-453, MDA-MB-361) were treated with trastuzumab for long durations[18]. This suggests that alternate ErbB family dimers, such as ErbB-1/ErbB-1 and ErbB-1/ErbB-3 dimers, could possibly circumvent trastuzumab-induced blockade, and favor growth and survival of resistant tumors. Moreover,

**TABLE 1**
**Novel Therapeutic Strategies for Trastuzumab-Resistant Breast Cancer**

| | Target | Mechanism |
|---|---|---|
| Lapatinib | EGFR and ErbB-2 | TKI |
| MUC4 monoclonal antibody | MUC4 | Neutralizing antibody |
| Pertuzumab | ErbB-2 | Dimerization inhibitor |
| Neratinib | EGFR and ErbB-2 | TKI |
| BIBW 2992 | EGFR and ErbB-2 | TKI |
| Temsirolimus | mTOR | TKI |
| Everolimus | mTOR | TKI |
| Sirolimus | mTOR | TKI |
| AP23573 | mTOR | TKI |
| Perifosine | Akt | Plasma membrane translocation inhibitor |
| CP-751871 | IGF-1R | Monoclonal antibody |
| NVP-AEW541 | IGF-1R | TKI |
| IMC-A12 | IGF-1R | Monoclonal antibody |

in ErbB-2–overexpressing MCF10A/ErbB-2 and BT474 cells, TGF-β has been shown to activate ErbB-3 and, subsequently, the PI3K pathway by enhancing phosphorylation and translocation of ADAM17 to the cell surface. This results in an increase in ErbB ligand shedding and desensitization of these cells to trastuzumab[19]. Interestingly, from EGFR and HER3 receptor knockdown studies, HER3 has been shown to play a crucial role over EGFR in ErbB-2–positive breast cancer[20] and ErbB-3 signals preferably by heterodimerizing with ErbB-2[21]. Therefore, the promising approach to treat trastuzumab resistance would be to design monoclonal antibodies that can target dimerization of all the ErbB family members. Pertuzumab, a monoclonal antibody against ErbB-2, prevents heterodimerization of ErbB-2 and has shown a 6-month clinical benefit when used in combination with trastuzumab in patients who had progressed after trastuzumab-based therapy[22,23]. Also, multitargeted tyrosine kinase inhibitors, including ErbB-1/ErbB-2–specific inhibitors lapatinib, neratinib, and BIBW 2992, have shown significant efficacy in trastuzumab-resistant disease[24,25].

In addition to functional redundancy in the ErbB family of receptor tyrosine kinases, insulin-like growth factor-1 receptor (IGF-1R) is another type I transmembrane receptor tyrosine kinase that is activated by IGF-1 and a related growth factor, IGF-2. Up-regulation of IGF-1R is thought to be one of the possible contributors of trastuzumab resistance[26]. IGF-1R blockade mediated by IGF-binding protein 3 was shown to resensitize SKBR3 cells to trastuzumab. IGF-binding protein 3 is a carrier protein for ligands IGF-1 or -2, and alters their interaction with IGF-1R, thus blocking downstream signaling. Recently, a novel cross-talk between IGF-1R and ErbB-2 has been identified[23], whereby IGF-1R can heterodimerize and transphosphorylate ErbB-2 in SKBR3-derived trastuzumab-resistant cells. Blocking IGF-1R signaling by either antibody or tyrosine kinase inhibitors resensitized SKBR3-derived resistant cells to trastuzumab. These results suggest a possible role for the IGF-1R signaling pathway in trastuzumab resistance, and that IGF-1R inhibitors in combination with trastuzumab may help to improve the efficacy of trastuzumab. CP-751871, a monoclonal neutralizing antibody, and NVP-AEW541, an IGF-1R tyrosine kinase inhibitor, should be tested either in combination or sequentially with trastuzumab in the metastatic setting. Although IGF-1R seems to be an important predictor of trastuzumab resistance in cell culture models, unfortunately in clinical tumor samples, IGF-1R expression levels have yet to show significant correlation with response to trastuzumab in women with ErbB-2–positive, metastatic breast cancer[27]. However, this disparity between cell culture models and human tumor samples might be due to the inability of immunohistochemistry to detect active IGF-1R. Therefore, it is plausible that tyrosine-phosphorylated IGF-1R or the immediate downstream effector, IRS-1, expression levels could provide better predictive measurements than total IGF-1R protein.

Importantly, the c-Met (mesenchymal-epithelial transition factor) receptor is another type I transmembrane tyrosine kinase receptor that is thought to be a major contributor of trastuzumab resistance. Hepatocyte growth factor (HGF) binds and activates the c-Met receptor[28]. The c-Met receptor and its ligand HGF are often overexpressed in breast cancer, including a subset of ErbB-2–positive breast cancer[29,30,31,32,33]. The aberrant expression of c-Met receptor is correlated with decreased overall survival and poor patient prognosis[34,35]. Activated c-Met receptor has been shown to abrogate the growth-inhibitory effects of trastuzumab and protect ErbB-2–overexpressing BT474 and SKBR3 cells by inhibiting p27 induction[36]. However, inhibition of Met resensitizes BT474 and SKBR3 cells to trastuzumab[36]. Consistent with this finding is that trastuzumab treatment rapidly increases expression of c-Met in ErbB-2–overexpressing breast cancer cells[36]. This is important, as simultaneous overexpression of both c-Met and ErbB-2 has been shown to cooperate to promote cellular invasion, suggesting that tumors expressing both receptors may be more aggressive. Thus, it is certainly possible to predict that a subset of women with ErbB-2–positive breast cancer who are also overexpressing c-Met might benefit from a combined therapy targeting both ErbB-2 and c-Met receptors.

## Role for Loss of Negative Regulators

Negative regulators of downstream signal transducers activated by ErbB receptors are also critical components that have been implicated in resistance to trastuzumab. For example, the tumor suppressor phosphatase and tensin homolog (PTEN) is a negative regulator of the PI3K/AKT signaling pathway. PTEN, by removing a phosphate group from PIP3, prevents phosphorylation and activation of AKT and subsequently controls cell survival, proliferation, and growth. Loss or decreased expression of PTEN has been reported in ErbB-2–amplified breast tumors and is associated with poor response to ErbB-2–targeted therapy[37]. Concurrently, constitutive AKT kinase activity has also been shown to promote growth and proliferation of breast tumors[38]. ErbB-2–overexpressing BT474 breast cancer cells that have heightened PI3K/AKT signaling and reduced PTEN expression were shown to be sensitive to PI3K or mTOR inhibitors, and these inhibitors were able to reverse trastuzumab resistance both *in vitro* and *in vivo*[37,39]. These results suggest that loss/low expression of PTEN and subsequent high AKT kinase activity serve as predictors of trastuzumab response, and those inhibitors of the PI3K/AKT/mTOR signaling pathway need to be explored in combination with trastuzumab to prevent possible trastuzumab resistance. However, when tumor samples from trastuzumab-treated women were analyzed for PTEN and AKT status, the expression levels of PTEN and AKT did not significantly correlate with response to trastuzumab-based therapy, time to disease progression, or incidence of CNS metastases[40].

Yet another critical negative regulator of cell cycle progression is $p27^{Kip1}$, whose loss of expression has been implicated in trastuzumab resistance[38,41,42,43]. The $p27^{Kip1}$ binds to cyclin E either alone or in a complex with cyclin-dependent kinase 2 (Cdk2) and inhibits the catalytic activity of Cdk2 to prevent Cdk2 from adding a phosphate group to its substrate. Trastuzumab induces a G1 cell cycle arrest and apoptosis within the breast tumor by enhancing the association of $p27^{Kip1}$ with cyclinE/Cdk2 complexes, thus increasing the half-life of $p27^{Kip1}$ and preventing phosphorylation of $p27^{Kip1}$ by Cdk2 and subsequent ubiquitin-dependent degradation[42,44]. Decreased $p27^{Kip1}$ levels and increased Cdk2 levels have been reported in trastuzumab-resistant breast cancer[43]. Depletion of $p27^{Kip1}$ using either antisense or siRNA prevented trastuzumab-induced growth inhibition in ErbB-2–positive SKBR3 breast cancer cells. Conversely, overexpression of $p27^{Kip1}$ or preventing $p27^{Kip1}$ degradation using a proteasome inhibitor MG132 resensitized resistant cells derived from the SKBR3 cell line to trastuzumab. These results suggest that $p27^{Kip1}$ is a yet another crucial trastuzumab-resistant marker. Additionally, as described previously, up-regulation of IGF-1R could be responsible for trastuzumab resistance. The mechanism could be twofold, where increased IGF-1R signaling directly contributes to cell proliferation, but also has been shown to up-regulate the E3 ubiquitin ligase SKP2. SKP2 can ubiquitinate and promote the degradation of p27$^{Kip1}$, resulting in a loss of G1 cell cycle arrest and responsiveness to trastuzumab[45]. Moreover, the identification of p27$^{Kip1}$ protein stability regulators, such as SKP2, could prove to be very important for the development of novel targeted agents. However, p27Kip1 protein expression is not a predictive factor of response to trastuzumab-based therapy in patients with ErbB-2–overexpressing, metastatic breast cancer[46].

## Role for MUC4: Altered Receptor-Antibody Interaction

An emerging mechanism that could possibly contribute to trastuzumab resistance is altered interaction between ErbB-2 and trastuzumab. For example, elevated MUC4 expression is observed during acquired trastuzumab resistance[47]. MUC4, a membrane-associated glycoprotein, is a member of the mucin family that covers the epithelial surface, including mammary epithelia, and plays a major role in protection of epithelial cells. A soluble form of MUC4 is secreted into milk and is thought to protect the intestine of the neonate[48,49]. Additionally, MUC4 has two important functions: antiadhesion[50,51] and modulation of ErbB-2 signaling[52,53]. The ascites sialoglycoprotein-2 (ASGP-2) subunit of glycoprotein MUC4 directly interacts with ErbB-2 via an EGF-like domain, sterically hindering trastuzumab binding to ErbB-2[54,55]. This interaction results in increased phosphorylation of ErbB-2 at tyrosine1248, a major contributor to the oncogenic activity of ErbB-2. MUC4 activates ErbB-2, but does not affect the expression of ErbB-2[54,55]. Depletion of MUC4 using siRNA increased trastuzumab binding and sensitized the resistant JIMT-1 breast cancer cells to trastuzumab[47]. Thus, novel agents targeting MUC4 expression and/or function in combination with trastuzumab might prove to be advantageous in the treatment of resistant tumors.

## Importance of Notch Signaling

Recently, we have shown a novel cross-talk between ErbB-2 and Notch-1, another breast oncogene[56]. High expression of the Notch-1 receptor along with its ligand, Jagged-1, in breast cancer is associated with poor overall survival, including ErbB-2–positive breast cancer[57,58]. The Notch-1 signaling pathway plays a decisive role in determining the fate of the neighboring cells, as well as in tumorigenesis, by inhibiting differentiation and promoting survival and proliferation[59,60]. In mammals, there are four Notch receptors (Notch 1–4) and five ligands (Jagged 1 and 2, and Delta-like 1, 3, and 4)[61,62]. Notch receptors and ligands are type I transmembrane proteins. Notch receptors are heterodimeric and are synthesized as single polypeptide precursors in the endoplasmic reticulum, which upon fucosylation by O-Fut get transported to the Golgi. Atypically, Notch activation requires cell-cell contact for ligand binding and a series of three proteolytic cleavages (S 1–3)[63,64]. In the Golgi, the full-length polypeptide undergoes an S1 cleavage by a furin-like proteinase to form a heterodimer consisting of two subunits: the extracellular Notch-1 ($N^{EC}$) and the transmembrane Notch-1 ($N^{TM}$)[63,65]. Processed Notch is then transported to the plasma membrane, where binding of a ligand on an adjacent cell triggers a conformation change in the heterodimer, resulting in dissociation of $N^{EC}$ and transendocytosis of $N^{EC}$-ligand complex in the ligand-expressing cell[66,67]. As a result, the $N^{TM}$ undergoes an extracellular S2 proteolytic cleavage by a metalloproteinase (ADAM10/17)[67,68], followed by an intramembranous S3 proteolytic cleavage by the γ-secretase complex[67], releasing an active intracellular form of Notch, referred to as Notch intracellular (NIC). NIC, upon translocation to the nucleus, binds CSL (CBF-1/RBP-Jκ/Suppressor of hairless/LAG-1) transcription factors and displaces the corepressor complex (SMRT, SKIP, CIR, and class I or II histone deacetylases, SHARP, CtBP/CtIP) with a coactivator complex (SKIP, MAML1, and histone acetyltransferase PCAF, GCN5, or p300), activating the gene transcription of various target genes, such as transcription factors, Hey and Hes family members, which are effectors for cell fate determination[69].

Osipo et al. showed that overexpression of ErbB-2 in breast cancer cells (BT474, SKBR3, and MCF-7/HER2) decreased Notch-1 transcriptional activity, whereas inhibition of ErbB-2 by trastuzumab or a dual EGFR/ErbB-2 tyrosine kinase inhibitor similar to lapatinib increased Notch-1 transcriptional activity[56]. Furthermore, combination of trastuzumab with a γ-secretase inhibitor (GSI) or Notch-1 siRNA enhanced the growth-inhibitory effects of trastuzumab and induced apoptosis in sensitive cells, and reversed trastuzumab resistance in resistant ErbB-2–overexpressing BT474 breast cancer cells[56]. These findings suggested a critical role for Notch-1 signaling in ErbB-2–positive breast cancer in response to trastuzumab and in trastuzumab resistance. The precise mechanism by which Notch-1 is activated and contributes to trastuzumab resistance is being actively investigated to determine the role of Notch-1 in responsiveness to trastuzumab and in the development of the resistant phenotype. Recently, the importance of protein sorting, recycling, and ubiquitin-dependent degradation has been implicated in regulation of Notch-1 signaling[70,71,72]. Cellular context dictates the sorting of Notch-1 via endocytosis either to a degradation pathway or to an activation pathway. Results from our laboratory show that Notch-1 and its ligand Jagged-1 colocalize to the submembranous endosomes in ErbB-2–overexpressing SKBr3 cells (unpublished data). However, upon trastuzumab treatment, Notch-1 and Jagged-1 exit early endosome antigen 1 (EEA1)–positive vesicles and accumulate on the cell membrane**.** These results suggest a possible role of ErbB-2 in retention of Notch-1 and Jagged-1 within the endosomes inhibiting Notch-1 signaling, which may be reversed upon trastuzumab treatment. Moreover, it has been reported that the ErbB-2 promoter is positively regulated by RBPJkappa (CSL) and Notch-1[13]. This and our results suggest that there may be a negative feedback loop between ErbB-2 and Notch-1, where activated Notch-1 activates ErbB-2, which in turn inhibits Notch-1 activity. Therefore, it is possible that when we block ErbB-2 using trastuzumab, we disrupt the negative feedback loop, resulting in deregulated hyperactivity of Notch-1 and tumor progression. Activated Notch-1 could evade ErbB-2–targeted therapy and contribute to trastuzumab resistance in multiple ways (Fig. 1).

Notch-1 could either directly or indirectly regulate previously identified pathways that contribute to trastuzumab resistance. Some of the mechanisms that could promote trastuzumab resistance include up-regulation of MUC4[47], IGF-1R[26,73], c-Met[36], and/or EGFR-HER3 receptors[18], or down-regulation of tumor suppressors PTEN[37] and $p27^{Kip1}$[43]. Interestingly, Notch-1 has been shown to regulate some of these genes in non–breast cancer cells[74,75,76,77,78]. These results suggest that activated Notch-1 in ErbB-2–positive breast cancer may result in resistance to ErbB-2–targeted therapies by down-regulating PTEN and $p27^{Kip1}$, and up-regulating IGF-1R, MUC4, or c-Met, and thus promote tumor progression. Signaling pathways responsible for cell survival and proliferation are linked by multiple cross-talk mechanisms. Activated Notch-1 has been shown to phosphorylate and activate $p56^{Lck}$, which in turn phosphorylates and activates the PI3K-AKT pathway in T lymphocytes[79]. Moreover, $p56^{Lck}$ has been shown to be of positive prognostic value in estrogen receptor (ER)-negative and ER-positive, ErbB-2–positive breast cancers[80]. These results suggest that increased Notch-1 activity could activate PI3K-AKT signaling via $p56^{Lck}$. Thus, blocking ErbB-2 using trastuzumab will force the signaling networks to respond by activating the Notch pathway that could potentially increase cell survival and proliferation to promote tumor progression.

The tumor microenvironment is critically important for communication between tumor cells, the stroma, and the vascular endothelium. Notch-1 signaling promotes tumor growth in a bidirectional cross-talk between tumor cells and the surrounding tumor microenvironment. For example, the Notch-1 ligand Delta-like 4 is expressed on the surface of vascular endothelial cells and can activate the Notch-1 receptor expressed on the tumor epithelial cells to promote productive angiogenesis and subsequent tumor cell proliferation. On the other hand, Jagged-1 expressed on the tumor epithelial cells could activate the Notch-1 receptor on the vascular endothelial cells to also direct endothelial cell proliferation and angiogenesis[81,82]. Thus, increased Notch-1 activity observed upon inhibition of ErbB-2 may promote tumor growth and angiogenesis by potentiating cell-cell signaling between the tumor and its surrounding microenvironment.

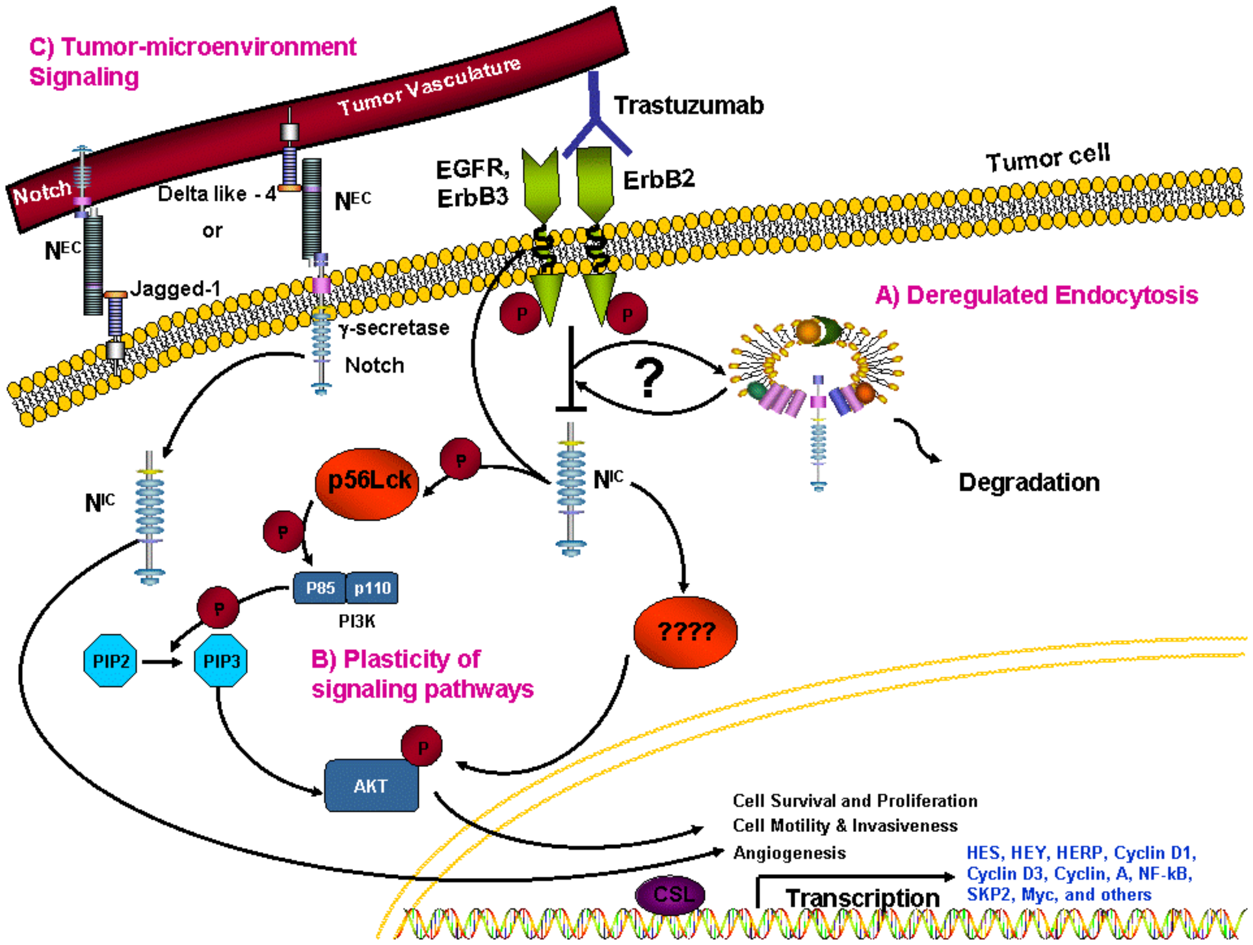

**FIGURE 1.** Possible mechanisms by which Notch is activated and contributes to trastuzumab resistance. (A) ErbB-2 may suppress Notch-1 signaling by retention of Notch-1 and Jagged-1 within endosomes, which may be reversed upon trastuzumab treatment. Activated Notch-1 could evade ErbB-2–targeted therapy by (B) activating the PI3K-AKT pathway via p56$^{Lck}$, and this functions to increase cell survival and proliferation due to plasticity of signaling pathways, or by (C) initiating a cross-talk between the tumor and its surrounding microenvironment, and promoting angiogenesis and tumor growth.

## CONCLUSIONS

ErbB-2 is a biomarker used to identify and treat breast cancer patients with trastuzumab-based therapy. This has provided for the dramatic increase in survival for women diagnosed with ErbB-2–positive breast cancer. However, resistance to trastuzumab is particularly problematic in metastatic disease and is one of the critical clinical challenges facing clinicians and researchers today. The use of genomic, transcriptomic, and proteomic arrays to understand molecular mechanisms of intrinsic or acquired ErbB-2–targeted drug resistance could provide tremendous information to identify patients that fail to respond to therapy or become resistant during therapy. Some of the molecular mechanisms for acquired resistance summarized in this review include overexpression of receptor tyrosine kinases (IGF-1R, Met), up-regulation of redundant ErbB family members, overexpression of MUC4, and loss of negative regulators (PTEN and p27$^{Kip1}$). These markers may also play a role in the development of intrinsic resistance to ErbB-2–targeted therapy; however, this area needs to be explored further. We present here a potential and novel biomarker of trastuzumab resistance: Notch-1. Activated Notch-1 could promote a survival advantage in response to ErbB-2–targeted therapies and contribute to resistance by regulating previously identified molecular markers of trastuzumab resistance, activating alternative signaling pathways, and potentiating cross-talk between the tumor and its surrounding microenvironment. Thus, a thorough investigation of the role of Notch signaling in ErbB-2–positive and other breast cancers would provide an evidential rationale of whether targeting the Notch pathway could prevent trastuzumab resistance, enhance, or restore sensitivity to trastuzumab.
